# Stigmatic limitations on reproductive success in a paleotropical tree: causes and consequences

**DOI:** 10.1093/aobpla/plx023

**Published:** 2017-06-15

**Authors:** Madhu Raina, Raman Kumar, Veenu Kaul

**Affiliations:** 1 Department of Botany, University of Jammu, Jammu, J&K 180006, India; 2 School Education Department, Government Higher Secondary School Parnalla, Jammu and Kashmir Government, Kathua, J&K, India

**Keywords:** Fluorescence microscopy, *Kigelia pinnata*, legitimate pollen, low fruit setter, ovule penetration, pollen limitation, self-incompatibility, thigmotropic stigma

## Abstract

Success in reproduction is subject to the successful initiation as well as successful completion of a chain of consecutive events starting from flower formation and ending with viable seed production. A pivotal role in this chain is played by the stigma which is the seat of pollen recognition and initiation of pollen–pistil interaction. An interesting feature of the family Bignoniaceae is the presence of thigmosensitive stigmas which open, close and re-open in response to touch. *Kigelia pinnata* bears a touch sensitive stigma and is a low fruit setter in Jammu and Kashmir (India). One possible reason might be pollen limitation coupled with reported self-incompatibility. However, not much is known about the mechanism of self-incompatibility in *K. pinnata* or of the role of its thigmotropic stigma. Carefully designed manual pollination experiments along with critical field observations revealed naturally deposited pollen load to be too low to cause permanent closure of stigma lobes. A strong relationship exists between the threshold pollen load on stigma, its permanent closure and fertilization. Of the various pollination treatments undertaken, fruits were formed only in open and manual cross pollinations when ∼9200 pollen of legitimate type is deposited on the stigma. Thigmosensitivity further limited the opportunity for the deposition of optimum pollen loads. Although, frequented by as many as seven different visitors, an average of 8 bats per night are available for as many as 30 trees which reflects a baseline deficiency of effective pollinators. This limitation in pollen and pollinator availability affects pollination success of this species and ends up with low fruit set. Fluorescence microscopy reveals successful germination and tube growth of pollen grains of both self and cross type but fruit-set is 100 % in cross-pollinated pistils only. Despite slower rates of ovule penetration and evidence of delayed fertilization, absolutely no fruit initiation occurs in self-pollinated pistils. This strongly points towards self incompatibility being late acting.

## Teaser

This article focuses on how thigmotropic stigmas play a role in limiting fruit set of plants in their non-native environment. Their pollination success requires optimum pollen transfer in terms of its quality as well as quantity and is dependent on efficient pollinator visitation. This study is particularly valuable both for horticultural purposes and general botanical research. This in turn can improve our ability to understand the behavioural response of exotic plants in non-native environments.

## Introduction

The Bignoniaceae with ∼900 species includes trees, shrubs and woody vines in the neotropics (Lohmann and Ulloa 2006; Rodriguez *et al.* 2016). However, those occurring in paleotropical regions are essentially arborescent (Machado and Vogel 2004). Considerable diversity in the reproductive biology (Amaral 1992; Stevens 1994) and pollination mechanism (Srithongchuay *et al.* 2008) of this plant group has been documented. Studies on the breeding system of neotropical taxa (Cope 1962; Jacob 1973; Taroda and Gibbs 1982; Bawa *et al.* 1985; Gibbs *et al.* 1999; Gribel *et al.* 1999) have revealed the prevalence of self-incompatibility. And the mechanism operative therein is largely late-acting (Gibbs 2014); a phenomenon delineated by the invariable failure of the selfed flowers to set fruit despite normal germination of pollen grains and growth of pollen tubes to the ovules (Seavey and Bawa 1986; Gibbs 2014). In published literature, it is also referred to as “ovarian sterility” or “pistillate sorting” (Sage *et al.* 1994). However, the phenomenon has been poorly understood (Gribel and Gibbs 2002). The euphilic flowers of this family display a wide spectrum of adaptations to different pollinator groups (Machado and Vogel 2004). The majority are pollinated by medium- to large-sized bees and some by hawkmoths (Vogel 1954; van Steenis 1977; Gentry 1990), birds (Porsch 1929; Weber and Vogel 1984) bats (Vander 1936/37, 1956; Gould 1978) and lemurs (Sussman and Tattersall 1976; Zihra 1998). All known paleotropical representatives in Old World are reportedly pollinated by Megachiroptera (Baker 1973; Vogel 1998) except tree-like *Musa* (Machado and Vogel 2004). Even when pollinators are abundant, most of the taxa are reportedly low fruit setters like *Catalpa speciosa* (Stephenson 1980), *Campsis radicans* (Bertin 1982) and *Tabebuia rosea* (Bawa and Webb 1984). Low fruit set in these taxa is generally attributed to high cost of fruit production (Ehrlen 1991) or to extrinsic causes like pollen limitation (Knight *et al.* 2005) and flower predation and/or internal factors like genotype and stored resources (Stephenson 1980, 1981; Weins *et al.* 1987). Such details on trees in India are limited (Tandon *et al.* 2003). For instance, only 2 of the 28 reportedly chiropterophilous plants (Subramanya and Radhamani 1993; Vikas *et al.* 2009) namely *Ceiba protandra* of Bombaceae (Nathan *et al.* 2005) and *Oroxylum indicum* of Bignoniaceae (Vikas *et al.* 2009) have been investigated in detail. In the latter, low fruit set is attributed to the poor pollination efficiency imposed by the thigmotropic stigma and a single visit of its legitimate pollinator *Cynopterus sphinx* Vahl.

*Kigelia pinnata*, a paleotropical species of Bignoniaceae (Machado and Vogel 2004), is native to tropical forests of Central Africa (Zjhera *et al.* 2004) where it is legitimately pollinated by *Micropteropus pusillus* Peters (Harris and Baker 1959). In India, *M. pusillus* is not found as per the Checklist of Valid Indian Bat Species (Talmale and Pradhan 2009). However, McCann (1931) and Subramanya and Radhamani (1993) have identified *Rousettus leschenaultia* Desmarest and *C.sphinx* Vahl. as the bat pollinators of this species from lowlands in India. Here, the species is reportedly self incompatible, pollinated by bats and either fruitless or showing very low fruit set (Chauhan 1995; Rana and Chauhan 1996; Rana 2009). Believed to have been introduced in India (Kumar 2007; Raina 2013, unpubl. data), the species is found abundantly in Southern parts of the country. It also grows in Jammu region of J&K state (Sharma and Kachroo 1981) along road sides, railway tracks and in rural areas. As such these trees do not form a huge component of the forests here. Instead trees occur in isolation at many places. A maximum number of eight trees were found growing at Ranbir Singh Pura (∼13 km from Jammu city); six at Gandhi Nagar; four, three, two and one at Bahu Fort, Jagti, Janipur and Nagrota, respectively, all in Jammu district of J&K. Against this a majority of 46 trees grow at 2 sites in the campus of University of Jammu and were, therefore, preferred for the study.

Except for few reports on phenology and anther dehiscence (Kumar and Kaul 2012; Raina and Kaul 2016), nothing substantial is published on the trees growing at Jammu (Kumar 2007; Raina 2013, both unpubl. data). These trees are prolific flower producers; the number of fruits formed, however, is not commensurate with that of flowers resulting in low fruit to flower ratio (Kumar 2007; Raina 2013, both unpubl. data). Low fruit set pattern in these trees growing in other parts of India like Agra are also on record (Chauhan 1995; Rana and Chauhan 1996; Rana 2009). Chauhan (1995) considers high temperature and pollen sterility to be the causes of fruitlessness at Agra. Later other factors like accumulation of large quantities of phenolics in stigma (Rana and Chauhan 1996) and, morphological differences in the stigmatic papillae (Rana 2009) have also been found to contribute to fruitlessness. Many workers attribute low fruit set to the presence of touch sensitive stigma and self-incompatibility (Stephenson 1981; Sutherland 1986; Primack 1987; Lee 1988). Reports on *K. pinnata* being self-incompatible are many (Canny 1973; Rana 2009) but the nature and type actually responsible are largely conjectural (Kumar 2007). Work carried out by Kumar (2007) on the same germplasm, however, could not ascertain the site of rejection with authenticity. Similarly, it was not possible to determine whether the same bat as reported by McCann (1931) or Subramanya and Radhamani (1993) effects pollination of *Kigelia* in Jammu.

Characterization of self-incompatibility in *K. pinnata*, therefore, required detailed tracking of pollen tube pathway following cross- and self-pollinations. It would also answer whether both the quality and quantity of pollen affect the stigmatic response. If they do, what could possibly be their extent and nature? And in what way does it have a bearing upon the extent of fruit set? We hypothesize three potential reasons to be responsible for low fruit set levels in Jammu: pollen/pollinator limitation, stigma sensitivity causing insufficient pollen deposition and self-incompatibility. To answer these questions we first evaluated the amount and nature of pollen deposition and its association with stigmatic movements and, second, we assessed the impact of pollen quantity and/or quality on fruit set vis-a-vis the breeding mechanism of the species. 

## Methods

### Study system

The present investigation was carried out on trees of *K.pinnata* growing at two sites in the campus of the University of Jammu, Jammu (J&K) India at 400 m altitude, 32°44′N latitude and 74°55′E longitude in sandy loam to clay loam soils. For this study 30 trees marked K1–K30 at one site near The Business School were randomly selected for detailed studies on stigmatic behaviour and breeding system. Trees flower twice a year—in February and August when average day and night temperatures vary between 18.4–6.1 °C and 41.5–26.4 °C respectively, and relative humidity ranges from 69 to 51.4 %. Differentiation of inflorescence primordia and opening of flower buds marks the beginning of its reproductive phase. These events commence in second and last week of February, respectively. In most of the trees, peak flowering is recorded during the months of April and May. Subsequently by the end of July the frequency of flowering decreases. In August, new inflorescences emerge which continue till October. However, the number of such inflorescences is considerably reduced relative to that formed in February.

### The stigma

#### Morphology and stigmatic movements

**Figure 1.**
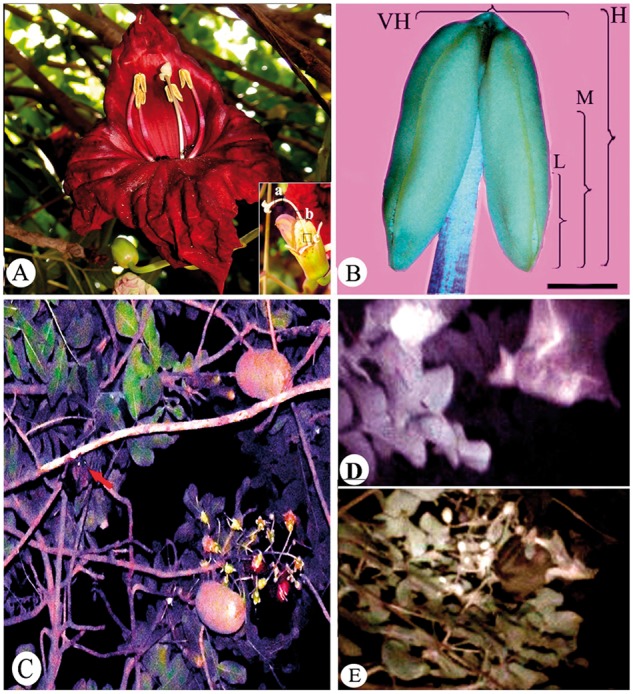
(**A**) Flower of *K. pinnata* showing fully unfolded stigmatic flaps and three levels of long style (∼7.86 ±0.21 cm) viz. top (a), middle (m) and base (b) examined for pollen tubes (inset). (**B**) Stereo-photomicrograph of an individual stamen of *K. pinnata* with the portions recognized as having low, L (eighth part), moderate, M (fourth part), high, H (half) and very high, VH (entire anther) quantities of pollen. Bar = 100 px. (**C**) A bat species (arrow) hovering around the flowers of *K. pinnata* and (**D**) about to visit it. (**E**) Bat foraging and pollinating the flowers. Note that it firmly holds lower corolla lip and consumes nectar by completely inserting its snout.

Stigma of *K. pinnata* is spathulate, bilipped, (Fig. 1A) wet and sensitive to touch. Nearly 1–1½ h post anthesis, the stigmatic flaps unfold. The exposed surfaces are papillate and highly sensitive to touch; gentle brushing forces their closure. This closure is transitory and the flaps re-open after some time. The cycle of folding and unfolding of stigmatic flaps continues till the stigma receives a threshold level of conspecific pollen. Therefore, the time taken to close and re-open in response to mechanical stimulus and pollen deposition were noted. For this purpose 50 flowers belonging to 5 trees were utilized; 10 for mechanical stimulation and 10 each for varying loads of self- and outcross-pollen, and of other *Bignonia* sp. and *Hemerocallis* sp.

#### Receptivity

To determine the exact time of initiation of stigma receptivity, three methods were employed:

(1) Peroxidase test

Since receptive stigmas are characterized by high levels of peroxidase (Dupis and Dumas 1990; Dafni and Motte 1998), these levels were determined by applying 3 % hydrogen peroxide to freshly unfolded flaps of excised stigmas (*n* = 5 per inflorescence per tree; number of trees = 3).

(2) Esterase activity

Like peroxidase, presence of esterases on the stigma is also related to receptivity. Hence, mature pre anthesis floral buds (*n* = 10 per 2 inflorescences per tree; number of trees = 2) were emasculated 2 h before anther dehiscence and then bagged. The mature unpollinated stigmas collected at 1, 2 and 3 h after anthesis were subjected to non-specific esterase test (Mattsson *et al.* 1974; Shivanna and Rangaswamy 1992). This was done to determine whether stigmas remain receptive up to 3 h after anthesis which happens to be the time of visitation of bats.

(3) Pollen germination test

Five flowers per inflorescence per tree (*n* = 10 trees) were randomly chosen one day before and on the day of anthesis, and tagged. As the stigmas unfolded, these were immediately subjected to manual pollination. One hour post-pollination, stigmas were examined microscopically for germination of pollen, if any. Those with germinating pollen grains on their surface were considered receptive.

### Threshold pollen load to initiate fruit set

For a complete understanding of stigma behaviour, three questions need to be answered: (1) What amount of pollen causes permanent closure of stigma? (2) Is this amount sufficient to cause fruit set? (3) Does quality or source of pollen also matter in (1) or (2) or both? A simple experiment was designed to answer these questions. Pre-anthesis mature flower buds were randomly selected (*n* = 40 per tree; number of trees = 5) and divided into two sets; Set I for self-pollination and Set II for cross-pollination. Equal number of flowers of each set was pollinated by different quantity/amount of pollen grains. Determination of the quantity of pollen to be utilized for deposition was made by dividing a single undehisced mature anther into eight equal parts. And pollen contained in the entire anther, half, fourth and eight parts of an anther were emptied and applied to the two flaps of different flowers. Loss incurred during cutting was not included in the pollen counts. It is pertinent to mention that each mature anther of *Kigelia* measures 12.5 × 0.8 mm^2^ and produces 100 153 pollen on an average.

Pollen for manual deposition was collected from mature undehisced anthers belonging either to flowers of same tree or those of different trees. These anthers along with their filaments were excised on the day of anthesis and stored at 4 °C for ∼10 h. Each anther was divided on a clean slide with the help of a clean sharp blade. The number of pollen grains per part per anther was estimated by scoring it separately from three random parts. An average of three replicates was taken as the pollen count per part per anther.

Flowers were also pollinated with pollen from less than one eighth of an anther (i.e. less than ∼9000 pollen grains). All the pollinated flowers utilized were bagged in transparent butter paper bags. Five days later, bags were removed to look for signs of fruit initiation.

As an initial measure of pollination success, amount of pollen sufficient to sire ovules should get deposited on the stigmatic surface. Stigma, in turn should also have enough surface area for successful landing of its own pollen along with pollen from other sources. Therefore, the surface areas of both the stigmatic flaps and pollen grains were measured. Surface area of the former was estimated by spreading and tracing the excised flaps of fully unfolded stigmas on a cm graph paper, and an average of 10 such scorings was taken. And that of latter using calibrated ocular and stage micrometers from freshly prepared acetocarmine mounts (*n*  =  15).

### Pollination experiments

Flowers of *K. pinnata* have a variety of visitors like wasps, bees, flies, ants, squirrels, sunbirds and bats, in morning and evening hours during different months. These seem to get attracted to the pollen and nectar. Visual observations find that the visitors while foraging frequently come in contact with the nectar and anthers; they rarely touch the stigma, raising doubts about their role in pollination. So to gain an insight into the type of breeding system operative in *K. pinnata* and the contribution of animal visitors to pollination success, flowers were subjected to the following pollination treatments in April, 2014:

1. *Control (natural or open pollination, OP):* Twenty-two inflorescences belonging to five different trees were tagged and kept for pollination to take place as it does in nature. These were monitored regularly from the time of flower opening to that of fruit maturation.
2. *Unassisted selfing/bagging (US):* Large number of buds in 20 inflorescences were bagged in transparent butter paper bags (18 × 28 cm^2^) to confirm unassisted selfing. Bags were used to exclude extraneous pollen and ensure that stigmatic lobes receive self pollen only. For this purpose, the bags were made porous with the help of a needle to allow free gaseous exchange. The size of the pore was small enough to exclude visitors of all types.
3. *Manual within-flower self-pollination (MSP):* Mature pre-anthesis flowers bagged prior to anthesis were self-pollinated. Pollen collected from their own freshly dehisced anthers was dusted on the exposed receptive surfaces of stigma to determine whether self-pollen is accepted or rejected and/or is capable of causing fruit set.
4. *Manual geitonogamous self-pollination (MGP):* Mature pre-anthesis flowers were emasculated before anthesis and pollinated with pollen collected from the anthers of other flowers of the same tree.
5. *Manual cross-pollination (MCP):* Mature pre-anthesis flowers emasculated before anther dehiscence were bagged. The stigmas which turned receptive later on were manually pollinated with pollen collected from flowers of adjacent trees to estimate extent of cross-pollen germination and also of resulting fruit set.
6. *Manual alien pollination (MAP):* Emasculated floral buds were bagged and receptive stigmas were manually pollinated with the pollen taken either from *Bignonia* sp. or from *Hemerocallis* sp*.* This was done to verify the role of foreign pollen on stigma sensitivity and their effect on fruit set, if any.
7. *Pollen transfer by insects (PTI):* Twenty inflorescences carrying pre-anthesis, mature and immature flower buds were bagged in porous transparent plastic bags. About 8–10 holes each of 2.5 cm diameter were made to allow the entry of all types of insects but exclude that of birds and bats. This would confirm their role, if any in pollination and successful fruit set.
8. *Apomixis (AP):* Thirty-five mature, un-open floral buds belonging to five trees were emasculated and bagged in butter paper bags (16.7 × 11.0 cm^2^) to test for non-pseudogamous apomixis, if any.

Twenty flowers from each treatment were kept undisturbed to look for signs of fruit initiation and development. The remaining were divided into two sets: those of one set were processed for fluorescence microscopy, and those of the other for light microscopy. The former was undertaken to observe pollen tube growth for which flowers harvested 24, 48, 72 and 96 h after pollination were fixed in Carnoy’s fluid (three parts of absolute alcohol and one part of acetic acid) for 24 h (Shivanna and Rangaswamy 1992). For light microscopic examination stigmas from flowers of each treatment were collected at regular 5 min intervals after pollination for 1 h and stained in Lewis’s (1979) stain.

### Visitor data

As already mentioned, *K. pinnata* receives a diverse array of visitors. Data on the number and behaviour of floral visitors were collected through visual observations for 7 days per month regularly from 21 trees. Three trees were monitored per day in the mornings (0600 and 1400 h, GMT) and late evenings (2330 h, GMT). These observations spread across 5 months from April to August. To monitor the foraging behaviour of each visitor correctly, they were temporarily designated as v1, v2, v3, v4, v5, v6 and v7 on the basis of their external appearance. Data were generated for each visitor on (i) frequency of visitation rates (ii) visit rate per hour and (iii) flower handling time and foraging strategy. A few specimens of each insect were trapped in net, anesthetized, mounted on paper pins and got identified with assistance from Entomology Lab, Department of Zoology, University of Jammu, Jammu, India. Similar monitoring was carried out for bats as well. Fifteen trees with intense flowering were monitored per night for this purpose. The time of visitation, mode of landing and foraging activity of bats were recorded by observing and tracking them in torch-, moon- and street lights using binoculars until they seemingly got satiated and stopped re-visiting the same flower. It is pertinent to mention here that *K. pinnata* exhibits flagelliflory; production of flowers on long rope-like branches, dangling beneath the crown. Two bats hovering about the flowers at night were trapped in net, anesthetized and brought to lab where their body parts were scanned for the deposition of pollen grains, if any. They were then preserved in 10 % formaldehyde (personal communication with Dr. Uttam Sakia, Officer Incharge, ZSI, Shillong) and were identified from Zoological Survey of India, Shillong, Meghalaya.

### Statistical analyses

All the data collected were recorded in Microsoft Excel (MS Office™ 2013). Standard descriptive statistics were performed on each quantitative parameter. Two-way ANOVA was conducted to detect the effect of type (self/cross) and amount (that from entire anther or half/fourth/eighth parts of anther) of pollen on time taken by stigmatic flaps to close.

Student’s *t*-test was applied to compare:

1. averages of time taken by stigma to close in response to pollen from one eighth of an anther of *Kigelia* and that from an entire anther of (a) *Hemerocallis* and (b) *Bignonia*.
2. average number of self pollen tubes (MSP) with those of cross pollen (MCP) at three stylar levels (a) top, (b) middle and (c) base
3. average number of pollen tubes entering (top) and leaving (base) the style in (a) self- and (b) cross-pollinated pistils and
4. difference between the number of ovules being penetrated by self and cross pollen tubes at three time intervals after pollination; (a) 48 h, (b) 72 h and (c) 96 h.

Of all the floral visitors of *Kigelia*, only bats forage, at the times of peak stigma receptivity, abundant pollen and nectar availability. While visiting, they repeat their foraging activity on different flowers and bring about effective pollination. In order to ascertain that bat visitation on the trees of *K. pinnata* affected the probability of fruit production, attention was focussed on their visitation rates to flowers.

The index of self-incompatibility (ISI) was determined as per Zapata and Arroyo (1978) for which data of controlled pollinations were utilized.

ISI = Fruit set after self‐pollination/ Fruit    set after cross‐pollination

Values ≥ 1 indicate self-compatibility; values greater than 0.2 indicate partial self-compatibility. Those less than 0.2, point towards predominant self-incompatibility and zero total self-incompatibility.

To illustrate the degree to which fruit setting propensity of the species is affected by an insufficient quantity/quality of pollen, one-way ANOVA was conducted to evaluate the impact of pollen source and quantity thereof on fruit set.

As per Pellegrino (2014), Pollen Limitation Index (PLI) was calculated as:

$$\text{PLI}=1-\left(\frac{P_{\text{o}}}{P_{\text{c}}}\right),$$

where *P*_o_ = fruit-set in open pollinated pistils, $P_{c}=\text{fruit}-\text{set in manual cross}-\text{pollinated pistils}$.

This index demonstrates the potential for fruit production when pollination is not limited. PLI values >0 represent pollen limitation.

### Photography

All outdoor photography was carried out with two digital photographic cameras—Sony Cyber Digital Still Camera (Model No. DSC-H100, China) and Sony Alpha 200 (Model No. DSLR-A200K, Japan). Photomicrography was carried out with the help of fluorescence microscope (Nikon Eclipse 80i, 551490, Japan). The bar given on all the photo- and stereo-micrographs (Nikon Digital Sight DS Fi2 Model No. SMZ 1500, Japan) represents 100 µm and 100 px, respectively.

## Results

*Kigelia* *pinnata* is a moderate-sized tree with grey-brown bark and a green, dense round canopy. Flowers are often borne terminally on thin rope-like long and pendulous branches. Inflorescence is a thyrse and ∼37.26 ± 4.35 (*n* = 15) are produced per tree per season. An individual thyrse measures upto 128 ± 2.29 cm and differentiates 15–69 flowers (40.46 ± 2.28; *n* = 20). Anthesis is nocturnal occurring around 1930 h. Usually, three to five flowers of an inflorescence open per night.

### Stigma receptivity

Anther dehiscence, marked by the formation of longitudinal slit, begins 7–8 h before anthesis (Raina and Kaul 2016). Approximately 1 h after anthesis, i.e. by 2030 h stigmatic flaps hitherto addressed gradually start unfolding and grow apart from each other. This unfolding is an indicator of stigma maturity. All such stigmas exhibit peroxidase and esterase activities and *in vivo* pollen germination at the time of anthesis; all positive indicators of receptivity. The stigmas remain receptive for 2 days after anthesis; whereafter receptivity declines. Folded stigmas do not respond to any test of receptivity indicating their immaturity prior to anthesis. The receptive stigma when pollinated manually with conspecific pollen supported about 40 % pollen germination when flaps made an angle of 30° with each other. The stigmatic flaps grow apart slowly and gradually. It takes about 1 h for flaps to align with each other at 180° (Fig. 1A). During this period of movement, pollen deposition induces stigma closure and compared with the percent pollen germination during partial unfolding of stigmatic flaps, a significant amount germinates upon complete unfolding (*t*_18_ =  13.31, *P* < 0.05). This indicates that complete unfolding of flaps is important for germination of maximum number of pollen grains. Stigmas remain receptive throughout that night till morning up to 0930 or 1030 h.

### Effects of various stimuli on stigmatic movements

#### Mechanical pressure

When the surface of a fully unfolded stigma is touched gently with any object, its flaps close immediately with the angle shifting from 180° to 0° within 6–8 s (6.62 ± 0.26; *n* = 10). The flaps re-opened 10–15 min later and did not close unless touched again.

#### Heterospecific pollen

Compared with the stimulus of touch, stigmas pollinated with pollen belonging either to an allied genus of the same family (*Bignonia* sp.) or to an unrelated genus of a different family (*Hemerocallis* sp.) take a longer time to close. They also re-open more slowly (Table 1).

**Table 1:** Response of freshly unfolded stigmatic flaps to pollen loads of varying nature and number.

Pollen load	Time taken (in seconds) by stigmatic flaps to close (A) and re-open (B) in response to pollination with			
	Self-pollen	Cross-pollen	Pollen from	
			*Bignoni*a sp.	*Hemerocallis* sp.
A				
<8th part	13.6 ± 0.75	14.4± 0.88	–	–
8th part	27 ± 1.03*	27.7± 1.02	30 ±1.2	34 ±1.07
4th part	29.8 ± 0.9	30.3± 0.83	–	–
Half	32.2 ±1.12	33.6 ± 1.07	87 ±1.0	38.9 ± 0.79
Entire	89 ± 0.92	90.5 ± 0.91	68 ± 0.7	111.1 ± 1.51
B				
<8th part	24 ± 0.84**	26.8 ± 0.42**	–	–
8th part	NR	NR	64.2 ± 1.23	86± 1.21
4th part	NR	NR	–	–
Half	NR	NR	81.2 ± 0.97	102.6 ± 1.87
Entire	NR	NR	104 ± 0.9	125.2 ± 1.87

* Mean ± S.E. (Range).

** In hours after pollen treatment.

NR: Never Re-open.

#### Conspecific pollen

**Figure 2.**
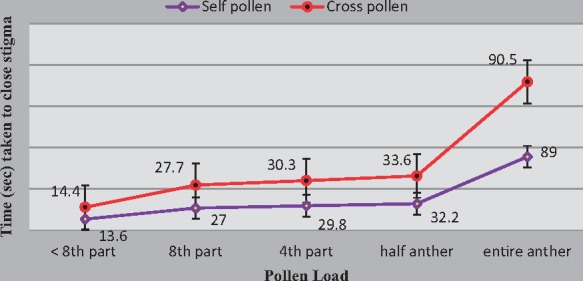
Comparative analysis of time taken (in seconds) by stigmatic flaps to close following pollination with self- and cross-pollen. Note that closure time varies significantly (*P* < 0.05) with pollen quantity but not with its source; self/cross (*P* > 0.05).

Stigmas with few pollen grains of *K. pinnata* close for ∼24 h and re-open thereafter. However, manual conspecific pollinations with very high (entire anther), high (half anther), moderate (one-fourth of an anther) and low pollen (one-eighth of an anther) pollen loads all resulted in permanent closure of the stigmatic lobes (Fig. 1B). The time taken by stigma to close varied significantly with amount of pollen received (*F*_3, 3_ = 7145 *P* < 0.05), but not with pollen type (self or cross; *F*_1, 3_ = 8.3917; *P* > 0.05) (Fig. 2). The mean time taken by the stigma to close largely depends on the amount of pollen deposited and is independent of its source.

The stigmatic flaps took an average of 27 s to close when pollen from eighth part of anther of *K.pinnata* was used. Contrarily the stigmas respectively take 68 and 111 s to close when pollen from an entire anther of *Bignonia* sp. and *Hemerocallis* sp. were applied. It may be noted that stigmas with pollen of *Bignonia*, allied genus of the family take lesser time to close (Table 1). The differences in the closure time are significant (*t*_18_ = 34.29 and 43.24; *P* < 0.05) indicating the importance of the pollen genotype.

On an average, the surface area of a fully unfolded mature stigma measures 1.03 cm × 0.44 cm with surface area of each flap being 49.5 mm^2^ and that of an individual pollen grain 0.0016 ± 0.0001 mm^2^. Theoretically, therefore, an average of ∼31 000 pollen grains can cover an entire stigmatic flap. But natural pollen loads were much smaller. Of 50 untreated flowers, 15 had zero pollen grains and the other 35 had an average of ∼122 pollen grains. This amount is also 4–5 times lower than the number of ovules [583.4 ± 37.96; *n* = 20].

### Pollination treatments

Of the eight pollination treatments, fruit set was recorded in only two; open (control) and manual cross-pollination (Table 2). Fruits were never set in the remaining six. Similarly, those pollinated with *Bignonia* and *Hemerocallis* pollen also aborted.

**Table 2:** Percentage fruit set in experimentally pollinated mature floral buds of *K. pinnata*.

S. no.	Type of pollination/ treatment	Number of inflorescences treated	Fruit set (%)	ISI	PLI
1.	US	20	0	–	–
2.	MG	10	0	–	–
3.	MAP	10	0	–	–
4.	PTI	20	0	–	–
5.	AP^†^	35*	0	–	–
6.	MSP	21	0	0	–
7.	MCP	25	100		0.986
8.	OP	21	1.318	–	

† Emasculated and bagged, without pollination.

* Number of flowers utilized for this treatment.

### Threshold pollen load

In flowers subjected to pollination with low pollen (eighth part containing ∼9200 pollen grains) treatments, stigmas, irrespective of the pollen source (self or cross) take lesser time to close their lobes permanently (Table 1). Fruit initiation in these occurred 5 days after pollination. The signs of fruit initiation are evident when the length of ovary (mean ± SE = 1.13 ± 0.03 cm) exceeds that of calyx (mean ± SE = 3.39 ± 0.10). While 17 out of 20 such flowers showed fruit initiation in manual cross pollen treatment, none were observed in manual self pollination. Similarly, in others where range of cross-pollen deposition on the receptive stigmas varied from moderate (fourth part) to very high (complete anther) none reopened. In ∼86.5 % of such flowers fruit formation initiated. However, pistils receiving pollen less than that present in the smallest i.e. eighth of an anther reopen their stigmatic flaps nearly 24 h after self- and 27 h after cross-pollination. Such pistils with inadequate pollen loads on their stigmas do not show any sign of fruit initiation. Stigma, style and ovary of these pistils sequentially turned black and shrivelled and eventually fell from the tree.

### Foraging strategy of visitors

Presence of large sized flowers, showy corolla, production of pollen grains in large quantity, copious nectar and musty-cabbagy odour appeal to a variety of pollinators (Table 3). Numerous giant (*Apis dorsata*) (v1) as well as small (*Apis cerana*) (v2) honeybees, wasps (*Vespa basilis*) (v3), ants (v4), sunbirds (*Nectarinia asiatica*) (v5), bats (v6) and three-striped squirrels (v7) frequent the flowers. Of these, bats, squirrels and wasps were the most frequent visitors (Table 3). While bats are nocturnal, all others are diurnal and visit flowers in the morning (0700–1315) and evening (1830–2045) h. With the exception of bats, none of the visitors affect pollination since at the time of their visitation either the anthers do not have enough pollen grains or even if they have, the stigmatic flaps are either closed or have lost their receptivity. This is strengthened by the results of the seventh pollination treatment (PTI) wherein no fruit set occurred. Bats, on the other hand, get attracted to foul odour emitted by the flowers at night. Before landing on the flower, we could observe bats usually hovering around flowers (Fig. 1C and D). Their visits start 1 h after anthesis, continue throughout the night and coincide with the times of stigma receptivity, pollen dispersal, peak nectar secretion and maximum odour intensity. On an average, a bat spends 7.6 ± 1.45 s (*n* = 30) on one flower and only occasionally more per flower. A single flower is visited ∼12 times per hour; each visit on individual flower per tree (*n* = 15) never exceeds 20 s. For foraging, an individual bat sits on the lower corolla lip, holds it firmly with its forelimbs and inserts its snout into the corolla tube brimming with nectar (Fig. 1E). Presumably, as it starts lapping nectar the upper lip of corolla carrying dehisced anthers is pulled down under its weight. With the result the dorsal side of its body comes in close contact with the anthers and gets laden with pollen grains. On visiting another flower of the same or different tree, the same bat repeats this activity during which the unfolded stigmatic flaps brush against the dorsal side of its body and get pollinated. In this way intra- as well as inter-plant pollen transfer takes place. That, a particular flower has been visited by bats is confirmed from the presence of claw marks on the flowers and fallen corollas.

**Table 3:** Comparative analysis of the data on frequency and foraging strategy of different visitors of *K. pinnata*.

Flower visitors	**Frequency per day/night**^†^	Foraging time (h)	**Visit rate per hour per flower**^♠^	Handling time (*n* = 20) (seconds) per flower	Foraging strategy
**I. Diurnal**					
**1.** Honey bees (*A. dorsata A. cerana)*	Numerous	1830–2045	3.9 ±0.59	21.7 ± 3.41	Forage pollen in evening and nectar in morning
**2.** Wasps (*V. basilis*)	Numerous	0700–1315	6.2 ±0.47	21.4 ± 2.56	Rob flowers of nectar and pollen. Also found roaming on extrafloral parts bearing nectaries
**3.** Ants	Numerous		5.6 ±0.391	192 ± 25.47	
**4**. Purple sunbird (*N. asiatica*)	16.1 ± 1.09	730	2.9 ± 0.291	11.2 ± 1.61	Consume left over nectar in the morning
**5**. Three-striped squirrel	6.14 ± 0.28	0800-1300	6.8 ± 0.436	6.6 ± 1.68	Hold floral buds within legs, open them with mouth and chew anthers
**II. Nocturnal**					
**6**. Bats	8.42 ± 0.59	2130 onwards	12.2 ± 0.75	7.6 ± 1.45	Forage flowers for nectar as well as pollen

† Monitored for 7 days/nights.

^♠^Monitored for 3 hours per day/night.

As per the reports from ZSI, Shillong the captured bats were identified as *Pipistrellus tenuis* (Temminck) and Asiatic Greater Yellow House Bat *Scotophilus heathii* Horsefield, both from family Vespertilionidae and sub-family Vespertilioninae. However, the former belongs to the tribe Pipistrellini and latter to Nycticeiini. Published literature about Chiropterans reveals that the two roost in the hollows of trees during day time and largely feed on insects during night. So, on the basis of their feeding habit, it is concluded that the two possibly are not the pollinator of *Kigelia*. Furthermore, no pollen of *Kigelia* was recorded on their body parts.

### Pollen–pistil interaction following manual cross- and manual self-pollinations and the pollen tube pathway

**Figure 3.**
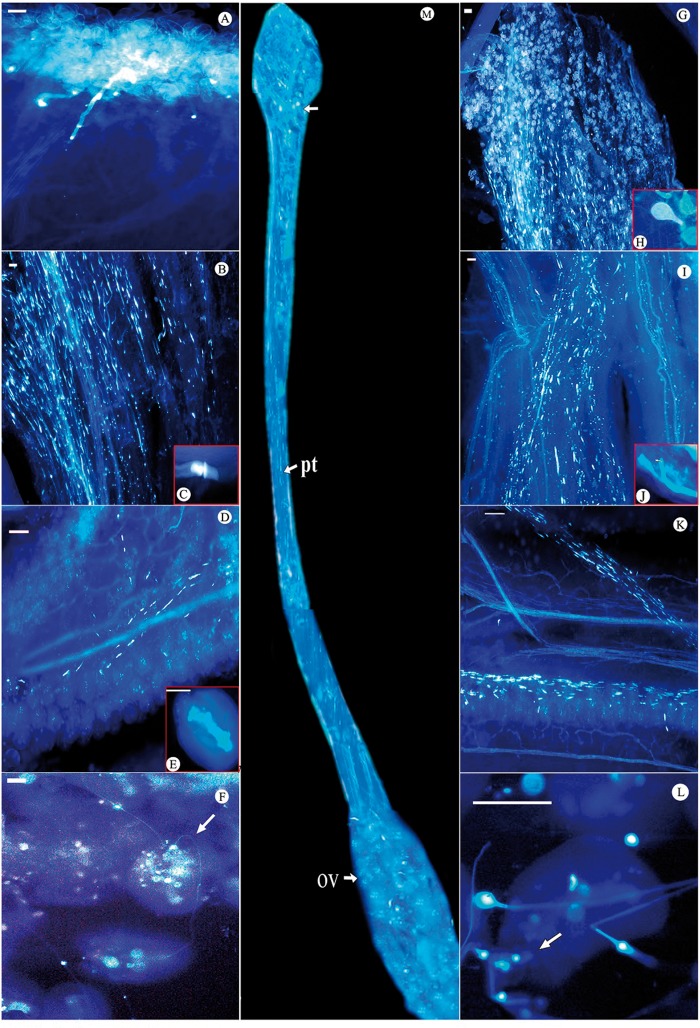
Fluorescence micrographs of self- and cross-pollinated pistils showing pollen–tube pathway. Pollen grain germination on the self- (**A**) and cross-pollinated (**G**) stigmatic surfaces and an enlarged portion thereof (**H**); in the mid-style (**B** and **I**) with brightly fluorescing callose plugs (**C**). Note the arrested growth at the base of the style in self-pollinated pistil (**J**) with arrow pointing towards the swollen pollen-tube tip. Pollen tubes enter the ovarian locule (**D** and **K**) containing cross-pollinated ovules with well formed embryo sac 43 h after pollination (**E**). Note the pollen–tube entering the micropyle (arrow) and penetrating ovules (**F** and **L**). (Scale Bar = 100 µm). (**M**) Fluorescence micrograph of reconstructed, cross-pollinated pistil of *K. pinnata* showing pollen tube pathway towards ovary.

Pollen grains, notwithstanding their source (self or cross) germinate within 13.3 ± 1.11 min of pollen deposition (Fig. 3A and G). Germination is prolific resulting in a mass of emerging pollen tubes which get attracted towards the stigmatic exudates (Fig. 3H) covering the papillae. The tubes grow down the surface of papillae, enter the style and thereafter, grow smoothly through the stylar canal. Pollen tubes of both the types reach the middle of the style within 24 h (Fig. 3B and I) developing a thin callosic wall and producing callose plugs at several intervals as they grow through the stylar canal to the ovary (Fig. 3C). However, a very subtle screening of the pollen tubes is noticed at three different levels circa 1cm down the stigma (top), in the middle, and at the base of the style. The number of both self- and cross-pollen tubes present on the stigma do not differ significantly (*t*_8_ =1.24; *P* > 0.05; Table 4). However, compared with cross-pollen tubes, growth of some self-pollen tubes stopped midway in the stylar canal. Tips of these pollen tubes get swollen due to callose deposition and never reach ovary (Fig. 3J). None of these abnormalities were observed in the growth of cross-pollen tubes such that significantly more outcross than self-pollen tubes reach the middle of the style (*t*_8_ = 3.30; *P* < 0.05). As pollen tubes of both the types approach ovary, the number of crossed ones reaching the base of style is significantly more (*t*_8_ = 1.88; *P* < 0.05). Inhibition in the growth of pollen tubes is also experienced within the pistil such that there were significantly more pollen tubes in the upper than in the lower ovary in both self (*t*_8_ =  6.03; *P* < 0.05) and cross (*t*_8_ =  2.18; *P* < 0.05) pollinated pistils.

**Table 4:** Pollen tube development scored at three different levels of style in *K. pinnata* after manual self- and cross-pollinations.

Treatment	Pollen germination (%) on stigma	Number of pollen tubes at three levels of the style		
		Top	Middle	Base
MSP	85	340.8 ± 1.61*	328.8 ± 2.33	302.0 ± 3.40
MCP	89	349.2 ± 1.67	343.4 ± 1.86	323.6 ± 2.24
		*t*_8_ = 1.24; *P* > 0.05	*t*_8_ = 3.30; *P* < 0.05	*t*_8_ = 1.88; *P* < 0.05

* Mean ± S.E.

Both self as well as cross pollen tubes enter the ovary 43 h after pollination (Fig. 3D and K). Following their entry, nearly all the self- or cross-pollinated ovules register a slight increase in size. However, this increase manifests in cross-pollinated ovules 12 h after pollination much before the entry of pollen tubes (Fig. 3E). Such ovules on an average measure 314.8 × 243.6 µm^2^ (*n* = 10) 12 h after crossing manually in comparison to 305.6 × 233.6 µm^2^ at anthesis; the difference though is not non-significant (*t*_8_ = 0.76; *P* > 0.05). A similar trend is observed after self-pollination. However, unlike ovules of manually cross-pollinated pistils, variation in the size of such ovules is detectable 43 h after pollination. And similarly their size at anthesis and 43 h after pollination does not vary significantly (*t*_8_ = 0.69, *P* > 0.05). Despite the large number of pollen tubes entering the ovary, only some make it to the micropyle (Fig. 3F and L). This is evident when only 1.2 % ovule penetration is recorded for manually selfed as against 7.6 % for manually crossed pistils 48 h after pollination (Table 5); the ovule penetration in crossed pistil being significantly higher (*t*_8_ = 11.1, *P* < 0.05). After 72 h post-pollination, the percentage of penetrated ovules significantly increases to 34.4 and 40.9 % for manual selfing and manual crossing, respectively (*t*_8_ = 15.5, *P* < 0.05). Similarly, 96 h after pollination about 94.1 % of the ovules exhibit cross-pollen tube entry against 80.5 % for selfed ones; the incidence of penetrated ovules being significant (*t*_8_ = 8.08, *P* < 0.05).

**Table 5:** Comparative analysis of number of penetrated ovules 48, 72 and 96 h following manual self- and manual cross-pollinations and t-statistics thereof.

**Hours (h) after pollination**	**Number of ovules penetrated following**		***t*_8_**
	**MSP**	**MCP**	
**48**	4 (1.2)**	26 (7.6)	11.1^*^
**72**	118 (34.4)	162 (40.9)	15.5^*^
**96**	261 (80.5)	288 (94.1)	8.08^*^

Each observation is an average of five readings.

* *P* < 0.05.

** Figures in parenthesis are % obtained from collective data on penetrated and non-penetrated ovules. However, data on the latter is not shown here.

### Fruit and seed set

**Figure 4.**
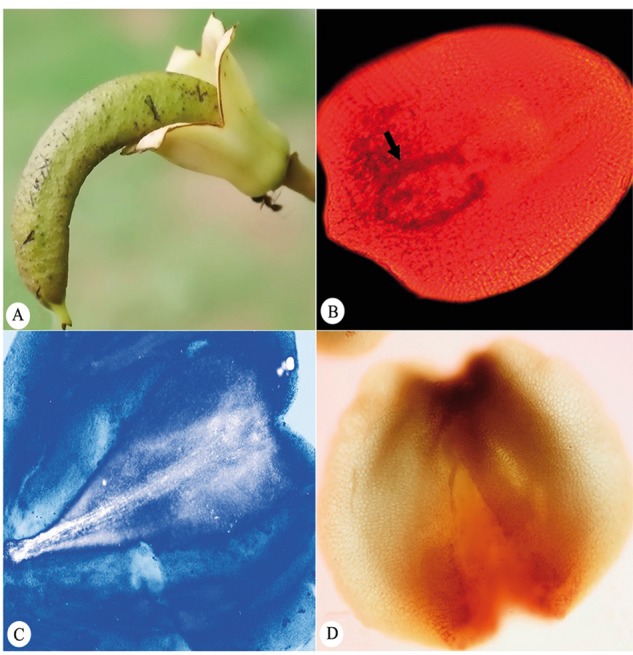
(**A**) A single immature fruit. Note the length of ovary exceeding that of calyx. (**B–D**) Seed from manually cross-pollinated fruits at different developmental stages; with a globular embryo (arrow) (**B**), an embryonal axis (**C**) and two cotyledons (**D**).

**Figure 5.**
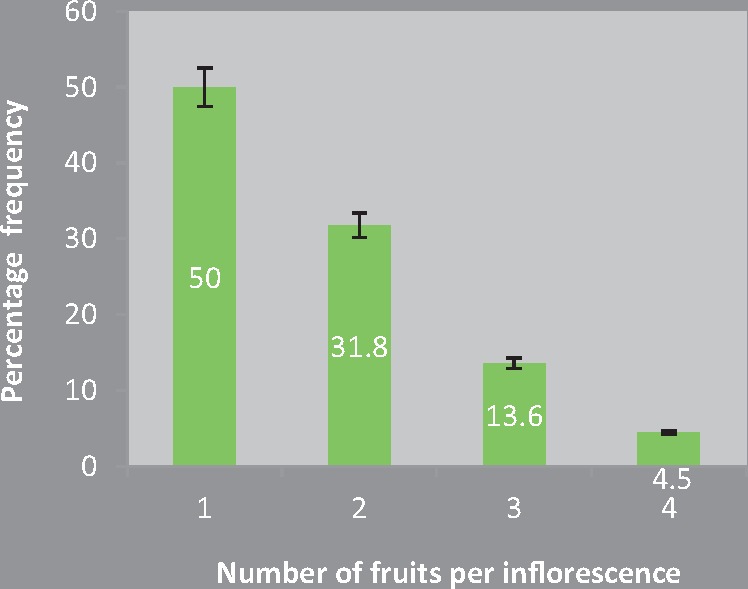
Percentage frequency of inflorescences with one, two, three and four fruits in non-experimental conditions.

Length of ovary exceeding that of calyx was taken as an indication of fruit initiation (Fig. 4A). In control (open-pollination), an average of 1.72 flowers per inflorescence transform into fruits. Although fruit development initiates in five to six flowers of an inflorescence the number maturing varies between 1 and 4. Nevertheless, the frequency of inflorescences with one fruit is the highest (50 %) and those with 4 is the lowest (4.5 %) (Fig. 5). Therefore, under non-experimental conditions fruit to flower ratio is extremely low ranging from 0.025 to 0.128 (0.063 ± 0.006; *n* = 17). Fruit set also varies with the pollination treatment; initiation is 100 % following manual cross-pollination (Fig. 4A). Results of one way ANOVA highlight the significant impact pollen source (open vs. MCP) had on percent fruit set (*F*_1, 4_ = 33.29, *P* < 0.05). Embryos microdissected from seeds of 5 and 9-day-old fruits and stained with 1 % acetocarmine were at globular stage (Fig. 4B) and with an embryonal axis (Fig. 4C) and two cotyledons (Fig. 4D), respectively. In contrast, no fruit was ever set in other pollination treatments except control. Therefore, compared with 100 % fruit set in manual cross-pollination, 0 % after manual self-pollination results in low ISI value and a mere ∼1.32 % in open-pollination works PLI at 0.986 (Table 2).

## Discussion

This article reports the first comprehensive study of stigmatic behaviour and its implication on the fruit set pattern and breeding system of *K.pinnata.* Like other tropical trees, *K. pinnata* flowers and sets seed every year (Joffe 2003; Rana 2009; Azu 2013). The structural organization of the flower and its essential organs is comparable to other bignons reported so far (Vander 1936/1937; Vogel 1969; Dobat and Piekert-Holle 1985; Machado and Vogel 2004). The tree has a long period of flowering extending from February to October and its spatio-temporal pattern of pollination activity remains the same during different flowering seasons. Its blooming pattern is characterized by the opening of relatively small number of flowers per night. This steady-state flowering (Gentry 1974) is common for bat pollinated taxa (Sazima *et al.* 1995, 1996; Bittencourt and Semir 2004).

Frequented by as many as six different visitors, only bats visit flowers of *K. pinnata* at the time of peak stigma receptivity, maximum pollen availability and abundant nectar. Their mode of visitation could result in successful intra- and inter-tree transfer of pollen grains from anthers to stigmas. Other visitors largely forage either for nectar or pollen or both at different time periods and do not affect pollination. This is primarily due to their smaller size such that at a particular point of time they do not establish any link or contact between pollen donation and reception structures. Further, at the time of their visitation anthers have little left over pollen or the stigmatic flaps are either closed or have lost their receptivity.

Success of any non-native plant depends on the mutualistic relationship it establishes with the resident biota (Richardson *et al.* 2000) which are required for pollination, successful fruit formation and seed dispersal (Parker 1997; Vanparys *et al.* 2008; Goodell *et al.* 2010; Gross *et al.* 2010; Rodger *et al.* 2010). Research on non-native plants has focused among other factors on their impact on pollination of co-flowering native species. However, little is known about the role pollination systems play in facilitating or constraining naturalization of immigrant plants (Traveset and Richardson 2006; Bjerkens *et al.* 2007; Castano *et al.* 2014). This study work is, in part, an endeavour in this direction.

The stigmatic flaps of *K. pinnata* close temporarily when pollen belonging to the same or different tree is deposited. This closure can be permanent provided the load is equal to or greater than the critical threshold. Results of this study put this threshold quantity at 9200 ± 1005.4 pollen grains. The time taken to re-open varies with the type of pollen deposited. Although several studies have shown that stigma closes permanently after being pollinated by cross pollen (James and Knox 1993; Stephenson and Thomas 1977; Stephenson 1979; Richardson 2004; Srithongchuay et al. 2010), others report permanent closure with self-pollen such as in *C.radicans*, *Anemopaegma laeve, Arrabidaea limae, Tabebuia impetiginosa* and *Jacaranda rugosa* (Yang *et al.* 2004; Pinheiro *et al.* 2009). Results presented herein indicate that, in *Kigelia*, stigmatic deposition of threshold self as well as cross-pollen loads commonly results in their permanent closure. However, in response to mechanical or touch stimulus or if they fall short of this critical threshold, the stigmatic flaps re-open after an interval of 24–27 h (Table 1). Presence of stigmas sensitive to mechanical stimulation with varied responses has also been reported in other members of Bignoniaceae (Newcombe 1922, 1924; Stephenson 1979; Bertin 1982; Peterson *et al.* 1982; Verma *et al.* 2001). Some show behaviour similar to *Kigelia* such as in *Proboscidea louisianica* (Thieret 1976), *C.speciosa* (Stephenson 1979), *Incarvillea emodi* (Verma *et al.* 2001), *C.radicans* (Yang *et al.* 2004) and *O.indicum* (Srithongchuay et al. 2010). In trumpet creeper (*C. radicans*), however, Newcombe (1922, 1924) showed that even after receiving a heavy pollen load, the stigmatic flaps open after initial closure but close permanently when touched second time. Newcombe interpreted second closing as a mechanism to provide a micro-environment suitable for pollen germination. In *Spathodea campanulata*, another bignon, the stigmatic lobes close permanently only after receiving both a threshold pollen load and pressure stimulus (Newcombe 1924).

In *Kigelia*, the stigma takes ∼13.6 and 14.4 s to close following self- and cross-pollen deposition, respectively. This time is quite longer than the foraging durations of bats which lasts an average 7.6 s, a feature common to several other members of Bignoniaceae (Bertin 1982). It is not known either as to how stigmatic flaps respond to pollen deposition in Africa or what is the foraging duration of the native bat pollinator there? Meanwhile, this gap of nearly 6–7 s, however, cannot reduce the likelihood of autogamous self-pollination nor does it reduce the chances of pollen from geitonogamous and heterospecific sources getting deposited on the stigma. High level of geitonogamy inducing permanent stigma closure is likely to diminish fruit set. The heterospecific pollen, on the other hand, clogs the stigmatic surface, reducing its availability to effective xenogamy (Pinheiro *et al.* 2009), delaying the germination of and fertilization by legitimate pollen (Ali 2013). Thus, the periodic closure and re-opening of stigma results in the stigma capturing doses of cross-pollen delivered. It is, therefore, likely that the bat’s first visits may result in deposition of cross-pollen and if it is optimum in fruit set. This is a common practice for other self-incompatible taxa of Bignoniaceae (Pinheiro *et al.* 2009).

In naturally pollinated flowers ∼30 % stigmas have no pollen while the other 70 % have an insufficient load (∼122) which manifests in extremely poor fruit set of 1.32 %. This means that despite bats making ∼12 visits per hour on the same flower, they are not able to deposit enough pollen to reach the critical threshold. By the time such stigmas re-open, there is a drastic reduction in floral rewards like pollen is completely discharged 12 h after anthesis (Raina and Kaul 2016), a gradual decrease occurs in the nectar volume and corollas have fallen. As such, the flowers are represented by the calyx cup and the pistil only. Lack of entire floral organization and of floral rewards sharply turns down the rate of re-visitation by the pollinator. Such stigmas fail to close for want of sufficient pollen which adversely affects their capacity to transform into fruits. Lack of fruit formation in all except two treatments points towards the following: (1) Non-pseudogamous apomixis is not operational, (2) Insects and visitors other than bats are pollen thieves and nectar robbers, (3) Pollen of auto-/geitonogamous source does not cause fertilization despite successful entry into ovules which strongly points towards the species showing bottlenecks in self-fruit set, (4) Pollen is not transferred to stigma without a pollinator. Fruit formation in control and manual cross pollination indicate that, (a) pollen deposition in control is less than the critical threshold; it could be of mixed (self/cross) type and very little of pure cross type, and (b) cross-pollen equal to or greater than the critical threshold is crucial to effect 100 % fruit set. Calculations based on these percentages yield zero ISI but high PLI values. While a zero value of ISI indicates complete or obligate self-incompatibility high PLI (0.986) points towards pollen limitation. This pollen inadequacy makes reasons obvious for its low fruit production in control. This might be a direct consequence of low baseline abundance of pollinators on the campus making the trees more prone to pollen limitation. Similar findings have been reported for other non-native plant species also (Wilcock and Neiland 2002; Knight *et al.* 2005; Aizen and Harder 2007). Low fruit production may be a part of the reproductive strategy of mass-flowering species of Bignoniaceae (Stevens 1994) because they produce relatively large fruits with numerous seeds (Pinheiro *et al.* 2009).

Subtle differences between self- and cross-pollinated pistils emerge at several points along the pollen–tube pathway in the progamic phase. Although the germination percentages of both are comparable yet the number of self- and cross-pollen tubes traversing the middle and base of the style differed. Self-pollen tubes experience an inhibition in their growth pattern by 4 % in the middle and 6.7 % at the base of the style. Once inside the ovary, the incidence of ovule penetration 48, 72 and 96 h after pollination reduces further. On the contrary, a significantly higher number of cross pollen tubes penetrate the ovules at these three time intervals. Whether this lag in rate of ovule penetration contributes significantly to failure of fruit set upon self-pollination remains to be ascertained. Marginal growth 43 h after pollination in self-pollinated ovules is indicative of a delayed event of fertilization having occurred. Embryo formation was, however, found in ovules of only cross-pollinated pistils 120 h after pollination. Despite more than 80 % ovules being penetrated by self-pollen tubes, lack of fruit initiation as well as embryo formation is a strong indicator of the involvement of some fruit aborting mechanism. Detailed histological studies are necessary to confirm the fertilization status of the selfed ovules and the extent of endosperm formation since ‘resting zygotes’ frequently occur in Bignoniaceae (Govindu 1950; Gibbs and Bianchi 1999). Further, early-acting inbreeding depression is least likely because ovaries would possibly not grow in size after self-pollination and penetration. Thus, it would not be pre-emptive to conclude that self incompatibility is operative in *K. pinnata* with the site of rejection being the ovule and mechanism, the late acting type. Similar reports by Gibbs and Bianchi (1993, 1999) are on record for *Tabebuia caraiba* and *T. ochracea*. On the contrary, in *Zeyheria montana* selfed ovaries did not show any swelling (Bittencourt and Semir 2004).

It can thus be inferred that presence of thigmosensitive stigma and self-incompatible nature of *K. pinnata* coupled with the pollen limitation are likely to tell upon its distribution as well as perpetuation over a long time range. Its future survival, therefore, appears to be dependent on the availability of efficient pollinators. Of its two pollinators—*C. sphinx* and *R. leschenaultia* reported in India—only the former is found in J&K (http://www.iucnredlist.org/details/6106/0 accessed on 18 January 2017). It has not been possible to trap and identify the bat which pollinates this species in Jammu. Since establishment and spread of non-native species are long term processes and do not rely on the success of reproductive season at a single site, the results of present work need to be extrapolated on trees of *Kigelia* growing in other introduced areas. This would be profitable to know whether the scarcity of pollinators and hence limitation of pollen varies across spatial and temporal scales.

## Conclusion

This study adds evidence to the hurdles of an out-crossing non-native plant to establish itself in the resident plant-pollinator network to set seeds. However, the reproductive assurance of *K. pinnata* documented here is poorly operating in the context of the species being non-native. A threshold number of pollen grains is required to be deposited on the stigma to enable permanent closure of its flaps. Evidence is presented here to show that its low fruit set levels in nature have been attributed to three related factors: pollen/pollinator limitation, stigma sensitivity and likely late-acting SI. The low fruit and low seed set in Jammu coupled with flowering season seemingly unfavourable for non-native pollinators are likely to tell upon its distribution as well as perpetuation over a long time range. We draw attention to the fact that fertilization in this species is conditional and subject to a critical pollen load deposition on the stigma. Similarly interactions within the ovules lead to inhibition of self seed formation. As a result seed set in nature is by cross pollen only. Despite slower rates of ovule penetration and evidence of delayed fertilization, absolutely no fruit set occurs in self-pollinated pistils. This is a strong indicator of the self incompatibility being late acting.

## Source of Funding

This study was supported by INSPIRE fellowship (No. DST/INSPIRE Fellowship/2014/124) by Department of Science and Technology (DST), Government of India, New Delhi. 

## Contributions by the Authors

Various parts of the experimental design have been conceived independently by all the three workers and collated for the final execution. M.R. drafted the article. M.R. and R.K. collected the data and analysed them. V.K. critically commented on the draft and approved the final manuscript.

## Conflict of Interest

No conflicts of interest.
